# Improving students' performance in geometry: an empirical evidence of the effectiveness of brainstorming learning strategy

**DOI:** 10.3389/fpsyg.2025.1577912

**Published:** 2025-07-01

**Authors:** Kazaik Benjamin Danlami, Yusuf Feyisara Zakariya, Bashir Balarabe, Sarah Bader Alotaibi, Tahani Mohammed Alrosaa

**Affiliations:** ^1^Department of Science Education, Ahmadu Bello University, Zaria, Nigeria; ^2^Department of Mathematical Sciences, University of Agder, Kristiansand, Norway; ^3^Deparment of Mathematics, Federal University of Education, Zaria, Nigeria; ^4^Department of Teaching and Learning, College of Education and Human Development, Princess Nourah Bint Abdulrahman University, Riyadh, Saudi Arabia

**Keywords:** brainstorming, geometry performance, instructional strategies, gender differences, mathematics education

## Abstract

**Background:**

The persistent challenges in geometry performance among secondary school students in Nigeria demand innovative teaching methods that extend beyond conventional strategies.

**Objective:**

This pretest and post-test quasi-experimental study investigated the effectiveness of brainstorming as an instructional approach to improve students' geometry performance.

**Methods:**

The research involved 140 students from two coeducational public secondary schools, with 73 students assigned to an experimental group taught using the brainstorming strategy. In comparison, 67 students were placed in a control group that received instruction through the conventional method. Data were collected using the Geometry Performance Test (GPT) with a reliability coefficient of 0.83 obtained using the test-retest method. The data generated were analyzed using descriptive and inferential statistics to address the research questions and test the study's hypotheses.

**Results:**

The results indicated that the experimental group significantly outperformed the control group in posttest scores, *F*_(1, 137)_ = 227.124, *p* < 0.001, with a mean score of 66.99 (SD = 9.17) compared to 46.76 (SD = 6.18) in the control group. No significant gender difference was observed in performance gains, *F*_(1, 34)_ = 1.609, *p* = 0.213, suggesting that brainstorming is an effective teaching strategy without gender bias.

**Conclusion:**

These findings show the potential of brainstorming as a powerful tool for improving students' performance in geometry with equitable effectiveness among male and female secondary students. Thus, we recommended that educators should integrate brainstorming into their mathematics classrooms as a proxy to close performance gaps among male and female students in mathematics and improve students' performance in mathematics. As this study was conducted in two public secondary schools in Kaduna State, Nigeria, we acknowledge that the findings are context-specific and may not be generalizable without caution.

## 1 Introduction

Geometry is a fundamental component of mathematics education, essential for developing critical spatial reasoning, problem-solving skills, and logical thinking. These skills are not only central to mathematics but are also vital for success in many STEM (Science, Technology, Engineering, and Mathematics) fields and everyday life. Geometry plays a crucial role in understanding the world around us, from the spatial organization of objects to real-world applications such as engineering designs, architectural structures, and even technological innovations. In sciences, geometry is indispensable for modeling spatial phenomena, including molecular shapes in chemistry and the vast structure of the universe in astrophysics (Rouvray, 1996). In engineering, geometric principles guide the design of mechanical systems and optimize construction processes, while architecture depends heavily on geometry to create aesthetically pleasing and structurally sound buildings (Ching, 2023). In applied mathematics, geometry supports complex computations in computer graphics and spatial algorithms (Dyn et al., 2020). Thus, the importance of geometry in both academic and practical contexts cannot be overstated.

Despite its significance, students often struggle with geometry, leading to persistent difficulties in performance. Research has shown that many students face considerable obstacles in comprehending and applying geometric principles, directly impacting their overall performance in mathematics (Atebe and Schäfer, 2008; Usman et al., 2020). One major issue is spatial reasoning, where students struggle with visualizing and manipulating geometric shapes in two-dimensional and three-dimensional spaces. They also face difficulty connecting abstract geometric concepts to visual representations, including interpreting diagrams and mentally constructing geometric objects based on textual descriptions. Additionally, students struggle to apply abstract geometric principles to real-world problems, making it difficult to translate everyday situations into geometric models. Many students rely on memorization rather than comprehension, limiting their ability to apply their knowledge flexibly and solve complex problems requiring conceptual understanding. The disconnection between geometry and other areas of mathematics further exacerbates these challenges, making it difficult for students to integrate geometric ideas with broader mathematical concepts. Several specific challenges contribute to these difficulties. Students often struggle with mentally rotating or transforming figures to solve geometric problems. Many find it difficult to interpret diagrams and figures, construct geometric objects from textual descriptions, and apply abstract principles to real-world contexts. Over-reliance on rote memorization rather than deep comprehension prevents them from flexibly applying knowledge to complex problems. Furthermore, geometry is often taught in isolation from other mathematical disciplines, such as algebra and measurement, making it challenging for students to apply geometric concepts across multiple areas.

Empirical evidence highlights that geometry performance has consistently been below average. According to Usman et al. (2020), only 34% of students demonstrated proficiency in geometry-based questions during standardized national assessments in Nigeria. Additionally, the West African Examination Council (WAEC) reports from 2017 to 2020 indicated that < 40% of candidates achieved satisfactory marks in geometry and measurement topics. These statistics underscore the urgency of addressing challenges in teaching geometry effectively. A major factor contributing to these problems is the widespread use of conventional lecture methods that do not enable students to engage in meaningful and active learning (Akaazua et al., 2017; Bot, 2018; Wahab et al., 2017). Ugboduma (2017) argues that traditional teaching methods are no longer sufficient to meet the changing needs of society. There is an increasing recognition of the need to explore alternative teaching strategies aimed at enhancing students' understanding and performance in geometry, a subject often perceived as challenging.

One particularly promising strategy is the brainstorming strategy, which encourages active participation from students. As noted by Halabiya (2022), this strategy engages students' cognitive abilities in problem-solving tasks, fostering an educational environment that prioritizes free thought and creativity. During brainstorming sessions, students express a wide range of ideas and perspectives, which not only helps in addressing specific geometrical problems but also cultivates critical thinking skills. This strategy helps students develop a deeper understanding of geometric concepts and encourages them to explore multiple avenues for problem resolution. Moreover, research conducted by Paulus and Kenworthy (2019) substantiates the efficacy of brainstorming as a learning strategy. Their findings indicate that brainstorming facilitates the generation of innovative ideas and enhances collaboration among students in group settings. The collaborative aspect allows students to learn from one another, exchange different viewpoints, and build on each other's contributions, ultimately leading to a richer educational experience.

Brainstorming has been widely recognized for its effectiveness in promoting active learning and academic performance (Mohammad, 2016; Owo et al., 2016; Unin and Bearing, 2016), though its specific impact on geometry remains underexplored, particularly in Nigeria. Thus, this study examines the effectiveness of brainstorming in improving student performance in geometry among upper primary school students. Additionally, it investigates whether gender differences exist in students' performance when a brainstorming strategy is used. By evaluating the effectiveness of brainstorming in the context of geometry education, this research provides valuable insights into innovative teaching methods that can enhance learning outcomes and contribute to improved mathematics performance.

To achieve these objectives, we raised and addressed the following research questions:

1. What is the effect of brainstorming as a teaching strategy on students' performance in geometry?
2. How does the brainstorming strategy impact the geometry performance of male and female students?

The findings of this study could be used to inform teaching practices, helping educators adopt strategies that involve all students, regardless of gender. This study focuses on geometry performance, a fundamental area of mathematics, to enhance our understanding of how collaborative learning techniques can promote equitable educational outcomes. This is especially important in regions where socio-cultural factors may restrict students‘ participation and performance. The remaining parts of this article provide an exploration of relevant literature, including an overarching theory for framing the hypothesized relationships between the research variables. The methodology section outlines the research design, including the approach used to investigate the effects of brainstorming on students' performance in geometry. Following this is the result section, where we present the descriptive and inferential statistics that form the basis for addressing research questions and hypotheses. We then discuss the findings in a separate section and highlight some implications before concluding the article.

## 2 Literature review

### 2.1 Challenges in geometry education

Students often face significant cognitive challenges when trying to master geometry. Key skills essential for success, such as spatial visualization, reasoning, and manipulating geometric shapes, can be difficult for many learners (French, 2004). A major obstacle in geometry education is the transition from concrete to abstract representations. Students often struggle to connect theoretical geometric concepts to real-world applications (Jablonski and Ludwig, 2023). Additionally, weak foundational knowledge from primary school mathematics further complicates these challenges, leaving students unprepared to tackle more advanced geometric ideas (Adolphus, 2011). Geometry is fundamental to mathematics education, enhancing spatial reasoning, problem-solving abilities, and critical thinking. It historically emerged from practical needs, such as land measurement (Jablonski and Ludwig, 2023), and its relevance has since expanded. Engaging with geometric concepts fosters essential cognitive skills, which are crucial for higher-level mathematical and analytical tasks. Despite its importance, students frequently struggle to grasp geometric concepts, impacting their educational trajectories and future career prospects. Beyond mathematics, geometry is significant across various fields, including architecture, engineering, computer science, and art. In architecture, geometric principles guide the creation of both functional and aesthetically pleasing designs. Engineers depend on geometry for structural analysis, while in computer science, it plays a crucial role in graphics and data modeling. Artists and geographers also apply geometric concepts to achieve visual balance and map spatial relationships, respectively (Kuzle, 2023). Therefore, geometry enriches not only mathematics education but also develops skills that are important across diverse disciplines.

Despite its importance, many students find geometry challenging. Research shows that students primarily struggle with spatial reasoning and visualization, which are vital for understanding abstract geometric concepts. Many learners have difficulty visualizing three-dimensional figures or making connections between geometry and algebra (French, 2004). Furthermore, assessments indicate that students often struggle to apply geometric concepts to real-world situations, such as using these ideas in problem-solving tasks or relating them to other mathematical forms. These difficulties are further intensified by inadequate prior knowledge and a limited understanding of fundamental geometric principles (Jablonski, 2024). The consequences of these challenges are significant. Students who struggle in geometry frequently perform poorly in mathematics overall, which can negatively affect their academic self-confidence and deter them from pursuing careers in STEM fields (French, 2004). Additionally, these struggles can limit students' problem-solving abilities, making it harder for them to tackle both academic challenges and real-life situations that require logical reasoning and spatial thinking. Addressing these challenges is crucial for ensuring that students not only improve their performance in mathematics but also develop skills necessary for success in various fields that rely on geometric knowledge.

Several factors contribute to the ongoing challenges in learning geometry. A significant issue is the inadequate foundational knowledge that many students bring to secondary school. This gap is often traced back to insufficient mathematical education during primary years, where foundational concepts should be established (Adolphus, 2011). Consequently, students may lack the skills and attitudes needed to engage with more complex geometric ideas. Psychological barriers, such as a fear of mathematics, also lead to disinterest and a lack of motivation to excel in the subject (Adolphus, 2011). Moreover, the quality of instruction plays a crucial role in students' understanding of geometry. Many geometry teachers have not received adequate training or professional development, undermining their ability to effectively teach complex concepts (Balarabe et al., 2024). The lack of access to appropriate teaching materials, such as visual aids and manipulatives, complicates students‘ learning experiences. Without hands-on activities, students often struggle to develop the spatial reasoning skills necessary for success in geometry (Markey, 2009). Moreover, students encounter difficulties in engaging with different representations of geometric concepts. Whether they are using photographs, 3D models, or real objects, the challenge of translating geometric ideas across various mediums hinders their ability to visualize and reason spatially (Jablonski, 2024). For example, students may find it challenging to grasp scale and perspective when working with images or 3D objects, which affects their understanding of geometry in real-world contexts. Integrating these real-world scenarios into teaching could enhance spatial reasoning and promote a deeper understanding of geometric principles (Jablonski, 2024).

### 2.2 Limitations of traditional teaching methods

#### 2.2.1 Rote memorization and passive learning

Conventional methods of geometry instruction predominantly emphasize rote memorization of formulas and definitions, which often hinders the development of a deeper conceptual understanding of geometric principles (Clements, 2003; Idris, 2005; Nazarovich and Kurudirek, 2024; Sinclair et al., 2016). While this memorization strategy allows students to recall relevant geometric properties during assessments, it limits their ability to apply these concepts in novel or unfamiliar contexts (Evans and Jeong, 2023; Nilimaa, 2023; Sukestiyarno et al., 2023). A promising approach to overcoming these inherent challenges is the implementation of active learning strategies. A study conducted by Juman et al. (2022) revealed that Grade 11 students in Sri Lanka struggled significantly with various aspects of geometry, including low motivation levels, inadequate foundational knowledge, and difficulties in effectively applying geometric theorems to solve problems. The researchers found that traditional teaching methods, characterized by lectures and passive learning environments, were less effective in directly addressing these issues. This prompted the suggestion that more interactive and hands-on learning approaches could enhance both student engagement and comprehension.

Active teaching methods, which include problem-solving tasks, interactive diagram drawing, and collaborative group work, have demonstrated a positive impact on students' abilities to visualize and apply geometric concepts effectively (Juman et al., 2022). For instance, when students engage in group discussions to solve complex geometric problems or when they utilize dynamic geometry software to explore properties and relationships, they develop a more nuanced understanding of the material (Harwood, 2023; Hwang et al., 2020; Nazarovich and Kurudirek, 2024; Tursynkulova et al., 2023). By fostering an environment of engagement and providing opportunities for authentic learning experiences, active learning strategies can effectively address many of the barriers students face in geometry education (Theobald et al., 2020; Vale and Barbosa, 2023). Furthermore, this approach not only aids students in grasping abstract concepts but also promotes the cultivation of critical thinking skills and problem-solving abilities that are essential for success in mathematics and other STEM disciplines (Mierluş-Mazilu and Yilmaz, 2024). A well-rounded geometry education is crucial (Harwood, 2023), as it lays the foundation for skills applicable in numerous fields, including engineering, architecture, physics, and even computer science.

However, geometry education faces significant challenges related to students‘ foundational knowledge, the quality of instruction, and their contextual understanding of geometric principles (Clements, 2003; Jones and Tzekaki, 2016). These obstacles can severely hinder students' success in geometry, reducing their capacity to excel in high-stakes scenarios that rely heavily on spatial reasoning and problem-solving abilities (Zhang et al., 2021). Addressing these issues is vital not only for enhancing student outcomes in mathematics but also for preparing them for success in the broader context of higher education and future careers. By adopting active learning strategies and implementing a more engaging, hands-on approach to geometry instruction, educators can empower students to navigate these challenges more effectively, fostering a deeper and more comprehensive understanding of the subject matter (Zakariya et al., 2016). In doing so, they will better prepare students for the complexities of both academic pursuits and real-world applications of geometry.

One of the most widely used teaching methods in geometry education is direct instruction (AlMutairi, 2015; Aung and Khine, 2020; Monye, 2016), where teachers present content through lectures and demonstrations. Using chalkboards, slides, or digital presentations, instructors explain geometric concepts, theorems, and problem-solving techniques while students take notes and follow along. Although this method ensures that all students receive the same structured information, it often places them in a passive learning role, limiting opportunities for engagement, exploration, and discovery. Without active participation, students may struggle to develop a deeper understanding of geometric relationships and their applications (Jones and Tzekaki, 2016; Sinclair et al., 2016; Theobald et al., 2020). Another prevalent approach is rote memorization, where students are required to memorize formulas, theorems, and definitions without always grasping their underlying principles. While this technique allows for quick recall of geometric rules during assessments, it does not necessarily lead to meaningful comprehension. Many students may successfully recite the Pythagorean theorem or calculate the area of a triangle but may find it difficult to explain why these formulas work or how they apply to real-world situations. Research indicates that students who rely solely on memorization often face challenges when confronted with unfamiliar problems that require conceptual reasoning and logical connections between different geometric ideas (Dhungana, 2021; Thagunna, 2015).

Traditional instruction often lacks opportunities for student engagement and interaction. Methods like direct instruction and textbook-driven learning tend to be teacher-centered, which limits collaborative learning, discussions, and problem-solving activities that encourage critical thinking (Malatjie, 2020). This passive approach can lead to disengagement, especially among students who learn best through hands-on experiences or visual demonstrations (Ang et al., 2021; Wurdinger and Bezon, 2009). Research suggests that interactive learning environments, where students actively explore and manipulate geometric concepts, lead to better retention and understanding compared to passive listening and rote exercises (Birgin and Topuz, 2021; Khormi, 2023; Mwangi, 2019; Siregar, 2024). Another significant limitation of traditional methods is their failure to relate geometric concepts to real-world applications (Gainsburg, 2008; Schoenfeld, 2013). Geometry is essential in fields like architecture, engineering, and design, yet conventional teaching strategies rarely highlight these connections (González and Herbst, 2006; Kaufmann, 2004). Consequently, students may view geometry as an abstract and theoretical subject rather than a practical tool for solving real-world problems (Hwang et al., 2020; Jurdak, 2016; Uyen et al., 2022). Without meaningful applications, students often struggle to see the relevance of their learning, which can diminish their motivation and interest in the subject (Noreen and Rana, 2019). The limitations of traditional teaching methods in mathematics education, particularly in geometry, necessitate the exploration of alternative instructional strategies. One such strategy is brainstorming, which provides an interactive and dynamic learning environment that fosters student engagement, creativity, and deeper understanding.

### 2.3 Brainstorming as an instructional strategy

#### 2.3.1 Theoretical foundations

The theoretical foundations of brainstorming are intricately linked to constructivist and social learning theories, establishing it as a powerful instructional tool for enhancing geometry education. Brainstorming, first introduced by Osborn (1963), serves as a collaborative strategy that fosters an open exchange of ideas, thereby encouraging creativity and facilitating deeper engagement with mathematical concepts. This instructional strategy is firmly rooted in constructivist learning theories, particularly those advanced by Piaget (1952). Constructivism posits that knowledge is not merely transmitted from teacher to student; rather, it is actively constructed through learners' experiences and collaborative interactions (Fosnot, 2013; Venter, 2001; Wells, 2002). According to Piaget, students achieve deeper understanding through active participation in problem-solving, which contrasts with traditional methods where information is passively received. Expanding on this idea, Vygotsky's social constructivism (Vygotsky and Cole, 2018) stresses the critical role of social interactions in cognitive development. They argue that learning occurs through meaningful dialogue and collaboration with peers and instructors. In this context, brainstorming effectively provides students with structured opportunities to exchange ideas, challenge each other's reasoning, and collaboratively build upon their collective knowledge. This dynamic interaction reinforces their conceptual understanding and promotes a more profound grasp of geometrical principles. Additionally, brainstorming is grounded in Bandura's social learning theory (Bandura, 1985, 2023), which underscores the significance of observation, imitation, and collaborative learning in the educational process. Bandura's research suggests that students enhance their problem-solving abilities by engaging in discussions with peers, which allows them to learn from shared experiences and gain insights from diverse perspectives. During brainstorming sessions, students are encouraged to articulate their thoughts, justify their reasoning, and critically evaluate different approaches. This active engagement not only cultivates cognitive skills but also fosters social skills essential for successful collaboration in mathematics and beyond. As such, brainstorming emerges as an ideal instructional method, seamlessly blending cognitive and social aspects of learning, making it particularly effective in the context of geometry education. Through this approach, educators can create an inclusive and dynamic learning environment that motivates students to explore, question, and deepen their understanding of geometric concepts.

#### 2.3.2 Benefits of brainstorming in geometry education

Brainstorming addresses several limitations of traditional instructional methods. Firstly, it promotes collaborative learning by encouraging students to work together to generate and refine ideas (Adedokun, 2022; Hamideh et al., 2023) unlike conventional teacher-centered approaches that often emphasize individual work and rote memorization, brainstorming creates an inclusive classroom environment where students actively participate in constructing knowledge. Research by AlMutairi (2015), found that students who engaged in brainstorming sessions exhibited improved engagement and problem-solving abilities, particularly in mathematics and geometry. By discussing different perspectives and approaches, students can clarify misconceptions, reinforce their understanding, and develop critical thinking skills (Bernardez and Alenton-Oracion, 2023). Secondly, brainstorming stimulates creativity, allowing students to approach geometric problems from multiple angles (de Vink et al., 2023; Hwang et al., 2020; Shchetynska, 2020). Unlike rigid instructional strategies that focus on procedural knowledge, brainstorming encourages students to explore unconventional solutions and develop original problem-solving techniques. Research by Silverstein (2013), shows that those students involved in brainstorming-based learning activities demonstrated higher levels of mathematical creativity and conceptual understanding compared to those who participated in traditional lecture-based instruction. This flexibility is particularly important in geometry, where visualizing spatial relationships and patterns requires innovative thinking. Additionally, brainstorming enhances students' ability to connect abstract geometric concepts with real-world applications (Chen, 2013; Mierluş-Mazilu and Yilmaz, 2024). Through group discussions and collaborative problem-solving, students can contextualize geometric principles, making them more meaningful and easier to remember (Hwang et al., 2020; Keshwan, 2014). A study by Costello (2021), found that incorporating brainstorming techniques in mathematics instruction resulted in greater retention and application of concepts, as students engaged in reflective thinking and articulated their understanding.

#### 2.3.3 Empirical support for brainstorming in mathematics education

Empirical studies highlight the effectiveness of brainstorming in mathematics instruction. Research by Obafemi (2024), found that brainstorming significantly improved students' problem-solving abilities and confidence in mathematical reasoning. Similarly, studies by Unin and Bearing (2016) demonstrated that brainstorming fosters motivation and active participation, particularly in collaborative learning environments. For instance, Obafemi's study indicated that implementing brainstorming techniques in classroom instruction enhanced not only students' problem-solving abilities but also their critical-thinking skills. The research revealed that students who engaged in brainstorming showed greater confidence in tackling complex mathematical tasks, as they were encouraged to explore multiple strategies and collaborate on solutions. Goos (2004), Raja (2018) and Walia (2012) highlighted that traditional teaching methods often limit student participation and hinder deep engagement with mathematical concepts. In contrast, brainstorming promotes a participatory learning culture where students feel motivated to contribute ideas without fear of judgment. This strategy aligns with the principles of problem-based learning, which advocate for active exploration and discussion to develop higher-order cognitive skills (Adedokun, 2022). Widiastuti et al. (2022), also examined the significance of brainstorming in enhancing students' critical thinking abilities. Their research showed that teachers effectively employed brainstorming techniques by posing challenging questions to stimulate students' critical thinking skills. The authors concluded that brainstorming is crucial during learning activities and advocated for its increased use to further develop students' critical thinking abilities.

Integrating brainstorming into geometry education offers numerous benefits that address the limitations of traditional teaching methods. Fostering collaborative learning, encouraging creative thinking, and facilitating a deeper understanding of geometric concepts, brainstorming aligns with contemporary educational theories and practices. It empowers students to take an active role in their learning journey and equips them with essential skills that extend beyond the classroom. Adopting interactive strategies like brainstorming can significantly enhance the educational experience, making learning both meaningful and enjoyable (Al-Khatib, 2012; AlMutairi, 2015; Tsai et al., 2020). Incorporating such innovative strategies will ultimately prepare students to navigate the complexities of geometry and other subjects with confidence and creativity (French, 2004; Sinclair et al., 2016). Thus, brainstorming stands out as an invaluable tool for transforming geometry education for the better. The teaching of geometry has long relied on traditional methods such as rote memorization and direct instruction. While these methods have been foundational, they often fail to engage students in deeper, more meaningful learning. Recent research suggests alternative instructional strategies can improve conceptual understanding, problem-solving skills, and spatial reasoning. Approaches like Van Hiele's phase-based learning (Abdullah et al., 2014; Abdullah and Zakaria, 2013; Aldiabat and Yew, 2024), problem-based learning (Anitha and Kavitha, 2022), and technology-enhanced methods such as GeoGebra (Dzulfikar and Turmudi, 2024; Nguyen et al., 2023) have gained popularity for their effectiveness in enhancing students' cognitive and conceptual development. While these strategies show promise, the search for innovative methods continues, and brainstorming is one method that is gaining attention.

### 2.4 Gender and mathematics education

Research on gender disparities in mathematics has produced a range of findings, reflecting the complexity of this issue. Some studies assert that there are no significant differences in mathematics achievement between genders when active learning strategies are implemented, such as collaborative brainstorming sessions (Ajai and Imoko, 2015). In contrast, other research highlights those sociocultural factors, including societal expectations and classroom dynamics, can significantly influence student participation and engagement, particularly among female students in specific educational settings (Ganley and Lubienski, 2016). To explore the impact of brainstorming strategies on gender disparities in geometry performance, it is essential to assess how this collaborative learning approach can effectively bridge participation gaps between male and female students. Research has shown that brainstorming, as an interactive educational technique, not only fosters active involvement but also facilitates the exchange of diverse perspectives and ideas (Băbut, 2021; HajAlizadeh and Khorasani Anari, 2016; Rickards, 1999; Yewande and Olawunmi, 2023). However, the dynamics of gender during brainstorming activities can be influenced by sociocultural norms. For example, studies conducted in certain Nigerian classrooms, particularly in northern regions, reveal that female students often exhibit lower engagement levels during group discussions due to entrenched cultural norms and possible biases that prioritize male voices (Atsuwe and Musa, 2021; Yewande and Olawunmi, 2023).

These sociocultural expectations can severely restrict girls' opportunities to benefit from collaborative learning strategies like brainstorming. By cultivating a supportive and inclusive classroom environment that actively encourages all students to contribute, brainstorming can potentially mitigate these barriers. This method may be especially beneficial for female students, as it provides a structured platform for them to articulate their ideas, receive constructive peer feedback, and build their self-confidence in mathematical reasoning. As students become more engaged in the learning process, research suggests that the performance gaps related to gender may diminish, particularly in subjects such as geometry, where visualization and spatial reasoning skills are pivotal to success. Consequently, this study aims to investigate whether the use of brainstorming as a teaching strategy can help equalize geometry performance across genders by addressing participation issues and promoting equitable involvement within the learning experience. We hypothesize that the implementation of the brainstorming teaching strategy in the context of geometry will not exhibit any gender bias, thereby fostering an inclusive educational environment that supports the success of all students.

## 3 Materials and methods

### 3.1 Research design

This research utilized a quasi-experimental design featuring a non-randomized control group to evaluate the effectiveness of a brainstorming strategy on student performance. Specifically, a pretest-posttest method was employed to measure the impact of this strategy on students' understanding and application of geometric concepts. The brainstorming strategy implemented in this study is a structured, collaborative approach where students work in small groups to generate a diverse array of ideas and solutions related to geometric problems. The decision to adopt a quasi-experimental design was driven by several practical constraints that precluded the random assignment of participants. In many schools across Nigeria, particularly in Kaduna State, classroom organization is often dictated by school schedules and administrative protocols established by educational authorities. Consequently, teachers typically assign students to fixed classes based on these guidelines. This practice complicates the ability to randomly assign students to either experimental or control groups, necessitating the use of a quasi-experimental framework for this study. The objective is to provide a thorough and equitable evaluation of the brainstorming strategy's effectiveness in enhancing students' performance in geometry.

### 3.2 Population and sample

The study population comprised all upper-basic eight (JSS 2) students in the Kaduna North Local Government Area of Kaduna State. For the sample, two coeducational public secondary schools were selected through a multistage sampling technique. The initial step involved purposive sampling to choose coeducational schools, deemed to better represent the overall student population. From nine coeducational schools, two were randomly chosen using simple random sampling. An intact class was selected from each of the two schools, leading to a sample size of 140 students 73 in the experimental group and 67 in the control group who were, on average, 13 years old. In the control group, the conventional method was employed, which is a prevalent instructional strategy in many Nigerian schools. This method involved direct instruction, where teachers presented geometric concepts, equations, and principles with minimal student participation. The teaching was primarily teacher-centered, with students passively listening and following the instructor's explanations. Opportunities for collaborative work or discussions among students were limited, as this approach focused on individuals completing their assignments or homework. In contrast to the brainstorming method, which emphasizes teamwork and group discussions for problem-solving, the traditional approach did not engage students actively in their learning, remaining largely centered on teacher-led instruction.

### 3.3 Research instrument

The Geometry Performance Test (GPT) was used as the primary data collection instrument to assess students' understanding and performance in geometry. The GPT was adapted from Akaazua et al. (2017), who previously validated the instrument in a study on geometry education in Nigerian schools. The test consisted of 30 multiple-choice questions, each with four options. The questions covered fundamental geometry topics such as angles, triangles, polygons, and mensuration, all of which are part of the upper basic eight curriculum. Examples of GPT items include: (1) Identify the number of sides in a hexagon (Knowledge); (2) Classify a triangle based on its sides and angles (Comprehension); and (3) Calculate the area of a shaded region in a composite shape (Application). To ensure content validity, the GPT was reviewed by subject matter experts, including two senior mathematics teachers and one university lecturer specializing in mathematics education. The experts assessed the instrument for alignment with the curriculum and ensured that the questions adequately measured the intended geometry concepts. Additionally, the GPT was piloted with 20 students from a school not included in the main study. The pilot test helped identify ambiguous or unclear questions, which were subsequently revised. The reliability coefficient of the instrument was estimated using the test-retest method, yielding a value of 0.83, indicating that the GPT was sufficiently reliable for this study.

### 3.4 Intervention: brainstorming strategy

**Figure 1:**
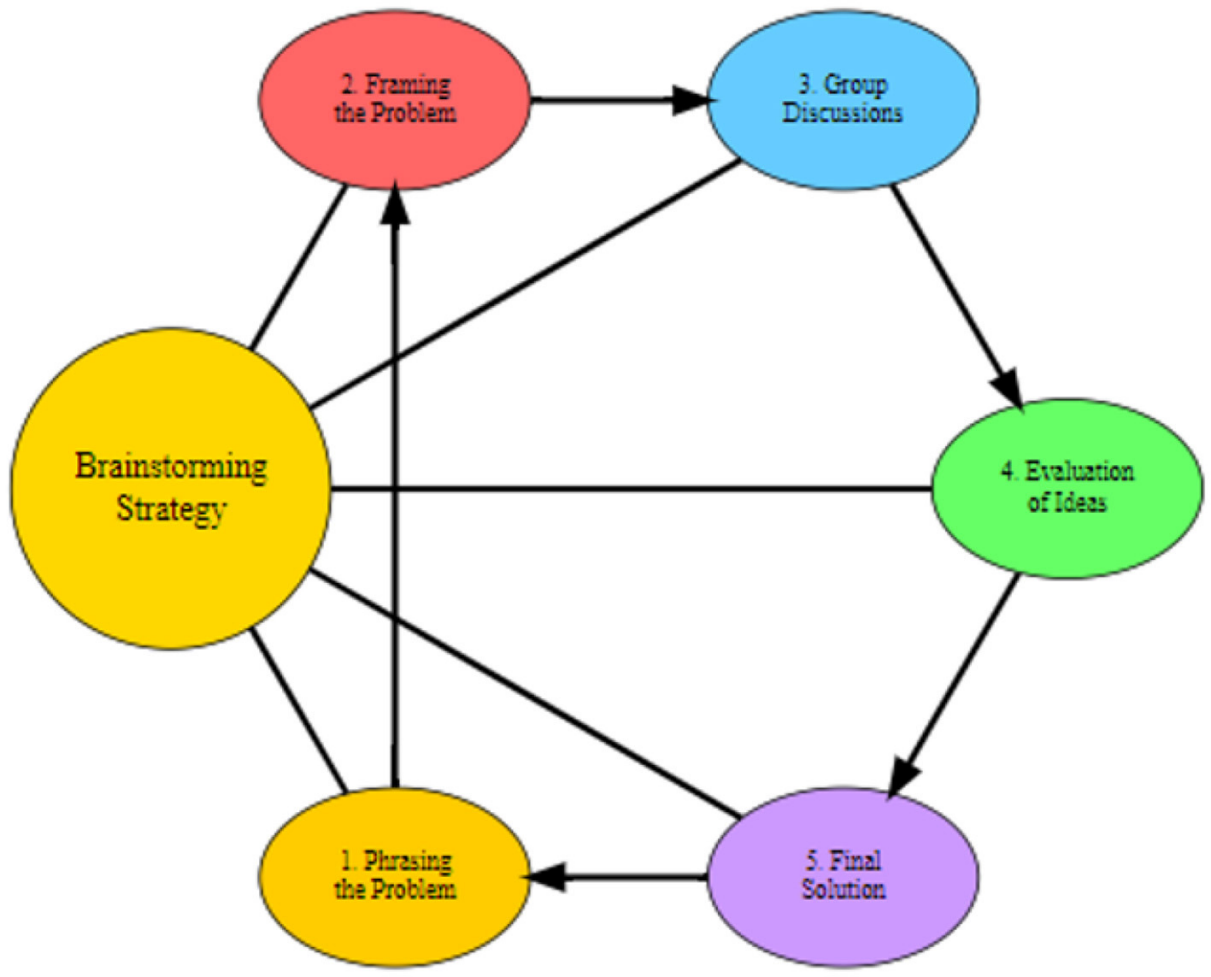
Brainstorming strategy process flowchart.

The experimental group was taught using the brainstorming strategy, which is a structured method designed to promote active participation, creativity, and critical thinking. The intervention lasted 6 weeks, with classes held once a week for 80 min. The lesson was designed based on the structured brainstorming strategy, following key steps such as phrasing and framing the problem, facilitating group discussions, evaluating ideas collaboratively, and working toward a final solution. These steps were specifically chosen to ensure active participation and idea generation in a collaborative environment. The steps involved in the brainstorming sessions were adapted from Mohammad (2016) and were designed to ensure replicability:

**Phrasing the Problem**: The teacher presented a geometry problem and discussed various methods of solving it. This step ensured that all students understood the problem before proceeding.
**Framing the Problem**: The teacher reframed the problem by breaking it down into key questions, helping students focus on specific aspects of the problem.
**Group Discussions**: Students were divided into groups of five. Each group engaged in discussions, where they generated ideas and explored potential solutions. Students were encouraged to share ideas freely without criticism to foster creative thinking.
**Evaluation of Ideas**: After the group discussions, the teacher facilitated a whole-class session where each group presented their ideas. Constructive feedback was provided, with students encouraged to critique solutions respectfully. The teacher then helped the class consolidate the correct ideas and correct any misconceptions.
**Final Solution**: Each group, with the help of the teacher, worked toward arriving at a final solution to the problem (Figure 1).

These steps were followed consistently to ensure a systematic application of the brainstorming strategy. The approach was designed to encourage collaboration, reduce anxiety associated with problem-solving, and enhance student engagement with geometric concepts.

### 3.5 Procedure for data collection

Before the intervention, a pretest was given to both the experimental group and the control group to evaluate their basic knowledge of geometry. This pretest was important to ensure that any differences in posttest scores were a result of teaching methods rather than initial differences in student knowledge. Both groups were taught for 6 weeks, with the experimental group receiving instruction on brainstorming strategies and the control group receiving conventional instruction. After the 6-week intervention, a posttest was administered to both groups. The pre and post-tests were identical, enabling a direct comparison of student performance before and after the intervention. The difference between pre and post-test scores was used to assess the effectiveness of the brainstorming strategy in improving geometry performance.

### 3.6 Data analysis

Data collected by the GPT were analyzed using the Statistical Package for Social Sciences (SPSS) version 25. Descriptive statistics, including mean and standard deviation, were used to summarize pre and post-test results. A two-way ANCOVA was performed to assess if any statistically significant differences existed in post-test performance between the experimental and control groups, controlling for pre-test scores. The factors considered were group (experimental vs. control) and gender, with post-test scores serving as the dependent variable and pre-test scores as the covariate.

## 4 Results

### 4.1 Data exploration

**Figure 2:**
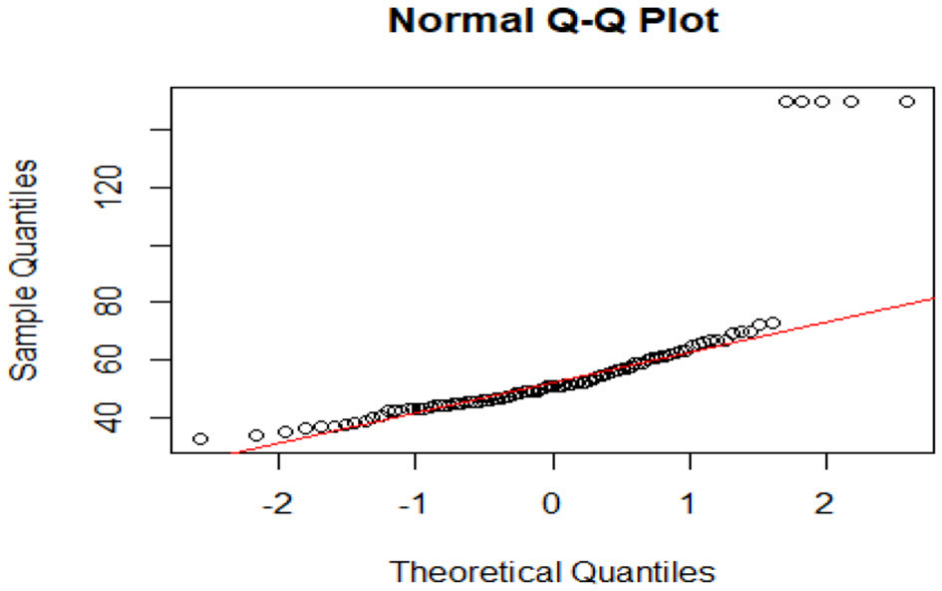
Normal plot graph.

Before exploring the research questions, an exploratory data analysis (EDA) was performed to examine normality, outliers, kurtosis, skewness, and missing data within the dataset. A Q-Q plot was generated for one of the variables (likely “variable_x”) to assess its normality. The plot indicated that most sample quantiles were closely aligned with the theoretical quantiles along the red line, especially in the central region of the distribution (Figure 2). However, notable deviations were present in the upper tail, where several points were significantly above the line. This implies that the distribution of the data is not perfectly normal and likely displays a rightward skew, which may affect the outcomes of statistical tests that presuppose normality.

Outliers were detected using both box plots and *Z-*scores. The Q–Q plot pointed to potential outliers in the upper tail, where points significantly diverged from the primary trend along the red line. *Z-*score analysis validated the existence of extreme values, particularly in “variable_y”, where two data points were identified as outliers, as they exceeded 1.5 times the interquartile range. Upon further examination, these points were confirmed to be legitimate data entries rather than errors, so they remained in the dataset.

To gain deeper insight into the shape of the data distributions, kurtosis and skewness metrics were calculated. While specific values are not detailed here, the analysis indicated positive skewness in at least one variable (likely “variable_y”), suggesting a right-skewed distribution. The analysis also pointed out the presence of excess kurtosis, indicating heavy tails in the data, which means the distribution featured more extreme values than would typically be expected in a normal distribution. Finally, a heatmap for missing data was created to evaluate the completeness of the dataset. The heatmap showed no missing values, as evidenced by the solid red color, confirming that all variables had observed data without any gaps needing imputation or other handling methods. In summary, the exploratory data analysis underscored departures from normality in the dataset, including skewness and possible outliers in certain variables. Nonetheless, no missing values were detected. These insights are crucial for informing the selection of suitable statistical tests and models for forthcoming analyses, particularly those that require normally distributed data.

### 4.2 Addressing the research question and testing the hypotheses

#### 4.2.1 Research question one: impact of brainstorming on geometry performance

The descriptive statistics for the pretest and posttest results of students in both the experimental and control groups.

**Figure 3:**
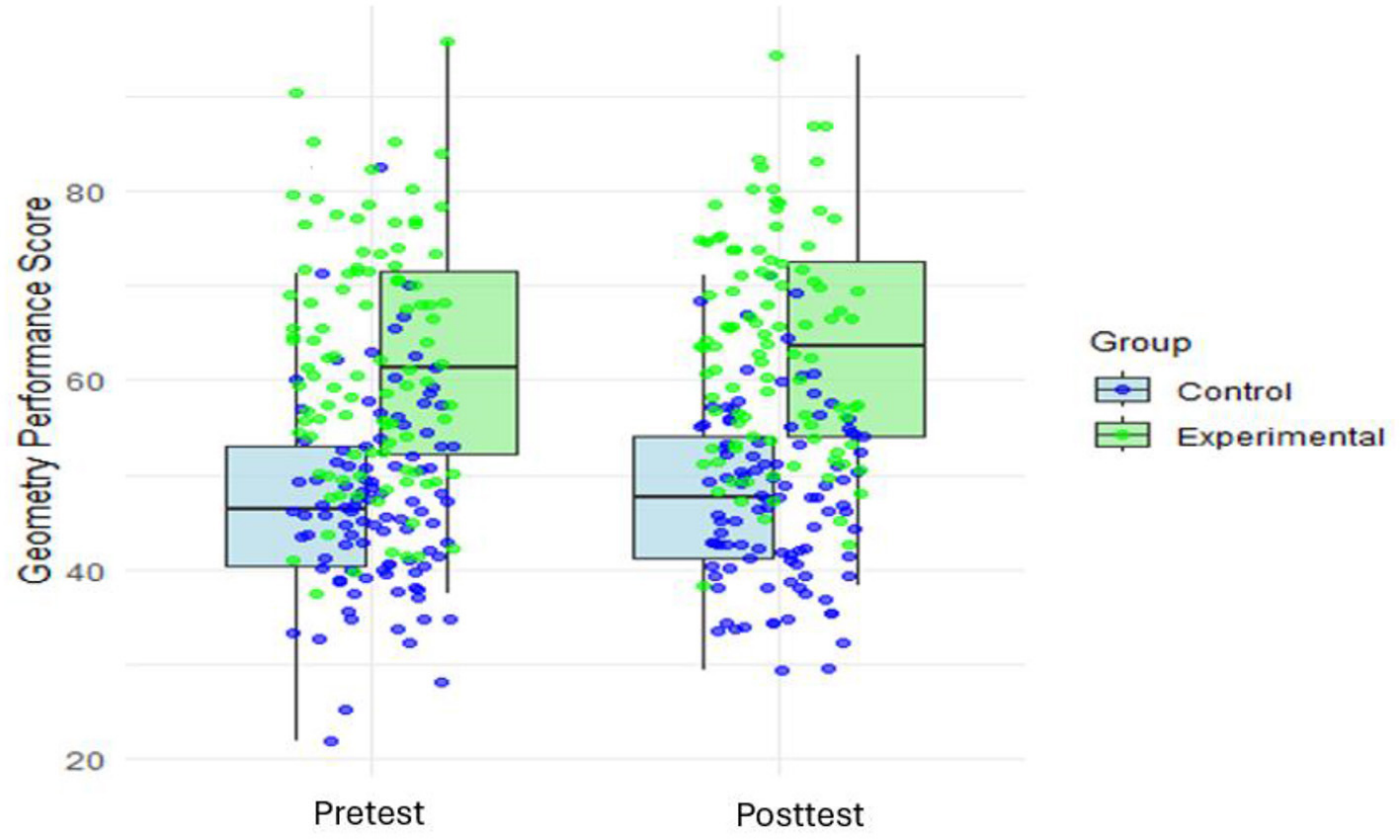
Pretest and posttest mean scores of control and experimental groups.

Figure 3 shows overlays of individual data points on box plots. This offers a comprehensive visualization of geometry performance scores across two groups (Control and Experimental) and testing conditions (Pretest and Posttest). This method allows for an insightful comparison between the two groups while simultaneously detailing the distributions, such as skewness, outliers, and kurtosis. Figure 3 depicts geometry performance scores on the y-axis, ranging roughly from 20 to 90, against the test variable on the x-axis, divided into Pretest and Posttest categories. For each test condition, two sets of box plots represent the Control and Experimental groups, color-coded in blue and green, respectively. Overlaying these box plots are individual data points, which allow readers to observe both overall patterns and specific characteristics of the data, making the visualization particularly effective for identifying trends and anomalies. Further, each box plot encapsulates the interquartile range (IQR) with the median distinctly marked within. The whiskers extend to include most of the data, except for the outliers, which are individually highlighted. This setup not only reveals central tendencies and spreads but also draws attention to anomalous scores that could be of particular interest. Such detailed visualizations are crucial for understanding the full scope of the data, as they allow researchers and readers to identify potential outliers or extreme values that may influence the overall distribution. In the Control group, the Pretest box plot shows a median score around 46, with a relatively narrow IQR, indicating a tight clustering of scores. There are no significant outliers, and the distribution appears symmetric, suggesting consistent performance among participants. The low median score suggests that the Control group started with lower baseline performance compared to the Experimental group. Transitioning to Posttest, the median increases slightly to ~48, indicating a modest change. This modest change aligns with findings from previous research suggesting that traditional teaching methods, without additional interventions, may not always sustain student performance over time (Nafis and Nasri, 2024).

Contrastingly, the Experimental group's Pretest median starts higher at ~60, significantly surpassing the Control group and indicating a stronger baseline performance. The IQR is wider compared to the Control group, reflecting greater variability in scores. A few high-performing outliers above the upper whisker reflect exceptional performance by some individuals. The distribution appears slightly right skewed, with the longer upper whisker indicating a concentration of higher scores. In the Posttest scenario, the median rises further to ~68, demonstrating improvement. The IQR narrows slightly, indicating decreased variability after the intervention. Despite this decrease in variability, the distribution remains somewhat right skewed, continuing to reflect a concentration of higher scores. Similar trends have been documented in studies exploring the effects of innovative pedagogical approaches on student achievement (Jacob et al., 2020), highlighting the potential of targeted interventions to elevate performance.

Comparing both groups, clear patterns emerge. In the Pretest, the Experimental group demonstrates significantly higher performance than the Control group, with a median score of 60 vs. 46. The Experimental group also exhibits greater variability, as indicated by the wider IQR. By contrast, the Control group shows a more uniform distribution with a narrower IQR and no significant outliers. In the Posttest, both groups show improvement, but the Experimental group experiences a more pronounced gain, increasing its median score from 60 to 68. The Control group's improvement is more modest, with the median rising from 46 to 48. The Experimental group maintains its higher performance level and continues to exhibit greater variability. Meanwhile, the Control group's distribution remains relatively stable, with minimal changes in skewness or outlier presence. This visualization generates several insights and potential research avenues. The pronounced improvement in the Experimental group provokes questions about the intervention's differential impact, possibly influenced by initial performance baselines or inherent learning predispositions. The strength of Figure 3 lies in its ability to juxtapose aggregate trends with individual data points, facilitating a diverse comprehension of performance dynamics.

#### 4.2.2 The corresponding hypothesis one

A covariate analysis (ANCOVA) was performed to investigate how group assignment (experimental vs. control) influences post-test scores while accounting for pretest scores. The findings are outlined in Table 1.

**Table 1:** ANCOVA results for post-test scores.

**Source**	**Type III sum of squares**	**d*f***	**Mean square**	***F***	**Sig**.	**Partial eta squared**
Corrected model	14,493.701^a^	2	7,246.851	113.583	< 0.001	0.626
Intercept	14,950.177	1	14,950.177	234.320	< 0.001	0.000
Pretest	8.626	1	8.626	0.135	0.714	0.000
Group	14,491.050	1	14,491.050	227.124	< 0.001	0.514
Error	8,740.942	137	63.802			
Total	475,907.548	140				
Corrected total	23,234.643	139				

a R^2^ = 0.624 (Adjusted R^2^ = 0.618).

The ANCOVA results demonstrated that the adjusted model was statistically significant, *F*_(2, 137)_ = 113.583, *p* < 0.001, with a considerable effect size (*R*^2^ = 0.624), indicating that the model accounts for 62.4% of the variance in post-test scores. The variable of primary interest, Group, had a substantial impact on post-test scores, *F*_(1, 137)_ = 227.124, *p* < 0.001. This implies that membership in either the experimental or control group explained a significant amount of the variation in post-test scores after adjusting for pretest scores. Conversely, the Pretest variable did not significantly predict post-test scores, *F*_(1, 137)_ = 0.135, *p* = 0.714, which suggests that pretest scores did not make a meaningful contribution to explaining the variance in post-test scores once group assignment was taken into consideration. This finding indicates that the differences seen in post-test scores arise from the group treatment rather than from initial differences in pretest scores. In summary, the ANCOVA findings validate the hypothesis that group assignment (experimental vs. control) significantly influenced post-test performance. At the same time, pretest scores were not notably impactful after accounting for group differences. This underscores the potential effectiveness of the treatment administered in the experimental group.

#### 4.2.3 Research question two: gender differences in performance

**Figure 4:**
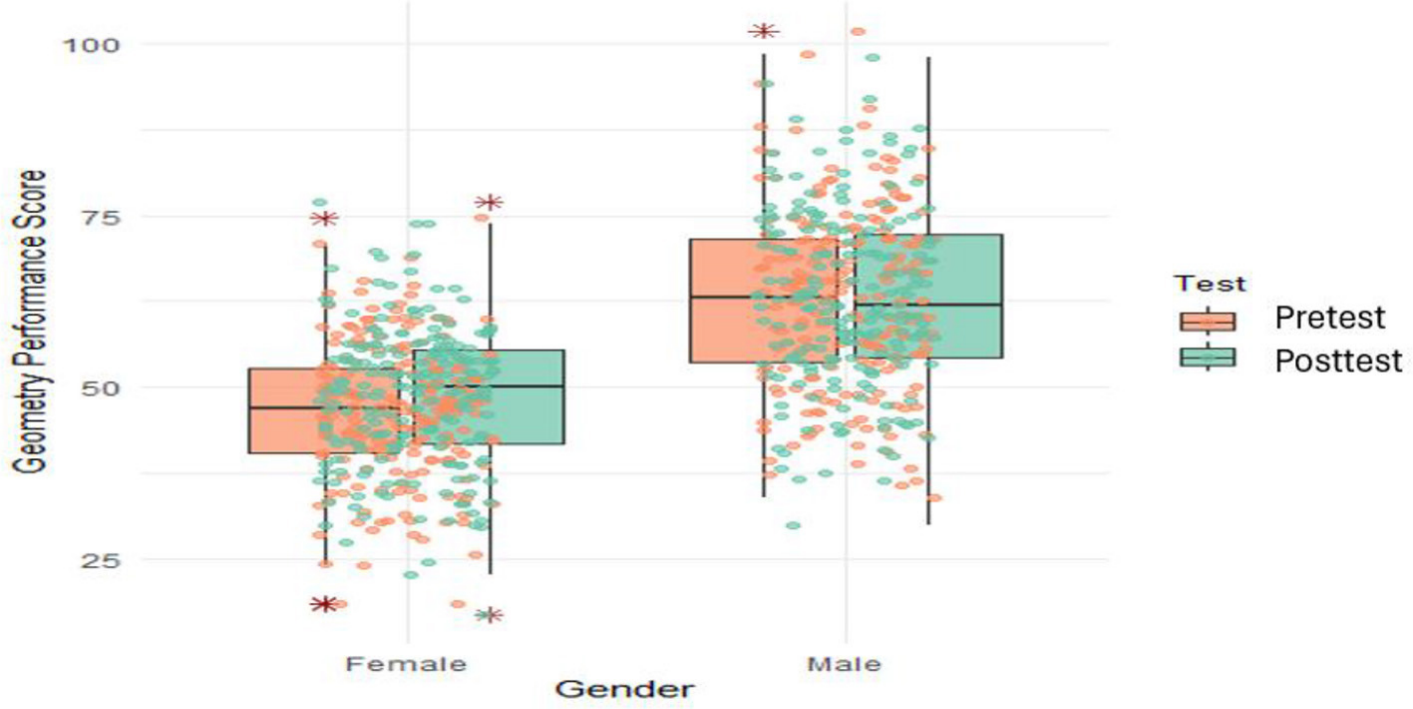
Comparison of pretest and posttest means by gender. * indicates outliers in the dataset.

To address the second research question, we explore the means and standard deviations of post-test scores of students taught using the brainstorming strategy and those taught using the conventional method. The descriptive statistics of this exploration are presented in Figure 4.

Figure 4 shows overlays of individual data points on box plots, offering a comprehensive visualization of geometry performance scores across genders and testing conditions. This method allows for an insightful comparison between groups while simultaneously detailing the distributions' nuances such as skewness, outliers, and kurtosis. Figure 4 depicts geometry performance scores on the y-axis, ranging roughly from 25 to 100, against the gender variable on the x-axis, divided into Female and Male categories. For each gender, two sets of box plots represent the Pretest and Posttest conditions, color-coded in green and orange, respectively. Overlaying these box plots are the individual data points, providing granularity to the analysis by showing the exact scores obtained by participants. This combination of box plots and individual data points enables readers to observe both the overall trends and the specific characteristics of each data point, making the visualization particularly effective for identifying patterns and anomalies. Each box plot encapsulates the IQR with the median distinctly marked within. The whiskers extend to include most of the data, except for the outliers that lie beyond, which are individually highlighted. This setup not only reveals central tendencies and spreads but also draws attention to anomalous scores that could be of particular interest.

In the Female group, the Pretest box plot shows a median score of around 46 with a narrow IQR, suggesting a tight clustering of scores. However, the presence of lower outliers indicates some significantly underperforming individuals. The distribution skews slightly right, given the longer upper whisker. Transitioning to the Posttest, the median elevated to 50, the IQR remains the same, hinting at relatively the same variability in the two test time points. This transformation suggests that the intervention had a positive impact on the Female group by improving consistency in performance. Contrastingly, the Male group's Pretest median starts higher at ~63, with a broader IQR indicative of greater variability. In the Posttest scenario, the median decreases slightly to around 62, maintaining a wide IQR but showing slight narrowing, suggesting some consolidation of scores. These findings indicate that the Male group maintained consistently high-performance levels, with the intervention leading to further improvements while retaining a degree of variability. Comparing both genders, clear patterns emerge. The Female group exhibits a larger relative gain, moving from a median of 46–50, whereas the Male group showed relative stability. Figure 4 generates some insights and potential research avenues. The pronounced improvement in the Female group provokes questions about the intervention's differential impact, possibly influenced by initial performance baselines or inherent learning predispositions. Meanwhile, the persistent variability within the Male group invites inquiries into subgroup dynamics contributing to high or low performance extremes. Additionally, understanding how interventions might be tailored to address specific needs could further reduce variability and elevate general outcomes.

#### 4.2.4 The corresponding hypothesis two

To investigate gender differences in student performance within the experimental group, a one-way Analysis of Covariance (ANCOVA) was performed. The pretest scores were included as a covariate to account for any initial variations. The results of the ANCOVA are presented in Table 2.

**Table 2:** One-way ANCOVA for gender differences in mathematics performance within the experimental group.

**Source**	**Type III sum of squares**	**d*f***	**Mean square**	***F***	**Sig**.	**Partial eta squared**
Corrected model	177.253^a^	2	88.626	0.981	0.385	0.055
Intercept	4,678.919	1	4,678.919	51.793	0.000	0.604
Pretest	52.773	1	52.773	0.584	0.450	0.017
Gender	145.321	1	145.321	1.609	0.213	0.045
Error	3,071.493	34	90.338			
Total	178,029.823	37				
Corrected total	3,248.746	36				

a R^2^ = 0.055 (Adjusted R^2^ = −0.001).

An Analysis of Covariance (ANCOVA) was performed to investigate the impact of gender on students' performance while controlling for pretest scores. The findings showed that the overall model was not statistically significant, *F*_(2, 34)_ = 0.981, *p* = 0.385, indicating that factors such as gender and pretest scores did not significantly predict performance outcomes. The intercept was found to be significant, *F*_(1, 34)_ = 51.793, *p* < 0.001, reflecting the anticipated performance score when all predictors are set to zero. Pretest scores were also not found to be a significant predictor of performance, *F*_(1, 34)_ = 0.584, *p* = 0.450, suggesting that pretest scores did not meaningfully affect post-test performance after accounting for gender. Furthermore, gender was not a significant predictor of performance, *F*_(1, 34)_ = 1.609, *p* = 0.213, indicating that there were no significant performance score differences between males and females when controlling for pretest scores. The group variable and the interaction between gender and group were excluded from the model due to either insufficient variability or were not relevant to the analysis. The error term had a sum of squares of 3071.493 with 34 degrees of freedom, resulting in a mean square error of 90.338. The *R*^2^ value was 0.055, meaning that the model accounted for ~5.5% of the variance in performance scores, while the adjusted *R*^2^ value was −0.001, implying that the model failed to address the variance in a meaningful manner when adjusted for the number of predictors.

## 5 Discussion and conclusion

The findings of this study show the effectiveness of brainstorming as a pedagogical strategy in improving geometry performance among junior secondary school students. The observable increase in post-test scores for the experimental group and the control group illustrates that brainstorming enhances comprehension and engagement with geometric concepts. The ANCOVA results revealed a statistically significant effect of group assignment on posttest scores, *F*_(1, 137)_ = 227.124, *p* < 0.001, with the experimental group achieving a mean score of 66.99 (SD = 9.17) compared to 46.76 (SD = 6.18) for the control group. This outcome is consistent with previous research advocating for the advantages of active learning methodologies. Studies by Ugboduma (2017) and Wahab et al. (2017) have similarly indicated that interactive and student-centered teaching techniques effectively address the shortcomings of traditional approaches while enhancing student performance in mathematical disciplines. The intervention demonstrated an equally positive impact across genders, further affirming its relevance as an inclusive teaching method. Descriptive statistics indicated comparable performance gains between male (mean gain = 40.18) and female (mean gain = 37.66) students, with no statistically significant differences in posttest scores, *F*_(1, 34)_ = 1.609, *p* = 0.213. These results concur with Ajai and Imoko (2015), who found that active learning strategies, such as brainstorming, facilitate equitable learning outcomes for both male and female students. This evidence challenges traditional beliefs positing that males are inherently more adept in mathematics, as suggested by Ganley and Lubienski (2016).

The outcomes of this study imply that the collaborative and supportive nature of brainstorming mitigates such biases, offering both genders meaningful engagement and opportunities to enhance their understanding of geometric concepts. While earlier research, including that of Goldenberg and Wiley (2011), cautioned against potential obstacles in brainstorming related to conformity pressures and groupthink, these issues were not observed in this study. The structured framework of the brainstorming sessions, incorporating elements such as problem formulation, group discussions, and evaluations, ensured that all students were encouraged to contribute and critique ideas respectfully. This design likely reduced the risks associated with group dynamics while amplifying the positive impact of the strategy on learning outcomes. The results align with constructivist and social cognitive theories of learning, which underscore the significance of active participation, social interaction, and collaborative problem-solving in knowledge acquisition (Bandura, 2023). By fostering an environment where students could openly express ideas, receive feedback, and collaboratively tackle challenges, brainstorming promoted critical thinking, creativity, and deeper cognitive processing. These attributes were evident in the significantly higher post-test scores observed in the experimental group. This study affirms that brainstorming is an effective instructional strategy for enhancing geometry performance. Its capacity to foster engagement, creativity, and equitable learning outcomes across genders renders it particularly valuable in overcoming the limitations of conventional methods. By incorporating brainstorming into mathematics classrooms, educators can cultivate inclusive and effective learning environments that empower all students to realize their full potential.

While the results of this research are encouraging, several limitations need to be noted. Firstly, the study employed a non-randomized sample due to logistical challenges within the school system, which restricts the ability to generalize the findings. The use of intact classes, though convenient, may have resulted in selection bias, as the experimental and control groups were not fully equivalent at the start. Future research should strive to use randomized control trials to provide more robust evidence of the causal link between brainstorming and enhanced geometry performance. Secondly, although the sample size of 140 students is adequate for statistical analysis, it hampers the generalization of results to the wider population of Nigerian students. Moreover, the study was conducted in a single local government area in Kaduna State, which may not represent the variety of educational contexts found throughout Nigeria. Cultural factors unique to Northern Nigeria, such as gender roles and classroom interactions, might have impacted the results, particularly regarding group involvement. Additional studies in other regions of Nigeria would be beneficial in determining whether the impacts of brainstorming remain consistent across diverse cultural and educational settings. Further, the study did not include a delayed post-test to assess the long-term retention of learning gains, which limits conclusions about the sustainability of the intervention's impact. The use of a non-randomized design limits the internal validity of the findings, as the experimental and control groups may not have been fully equivalent at baseline. Finally, the research concentrated exclusively on students' performance in geometry, neglecting other crucial factors such as students' attitudes toward mathematics, the long-term retention of knowledge, or the influence of teacher involvement on the success of brainstorming. Considering these factors could offer a more holistic perspective on how brainstorming affects learning outcomes.

## Data Availability

The raw data supporting the conclusions of this article will be made available by the authors, without undue reservation.
